# One‐year analysis of Elekta CBCT image quality using NPS and MTF

**DOI:** 10.1120/jacmp.v17i3.6047

**Published:** 2016-05-08

**Authors:** Satomi Nakahara, Masayuki Tachibana, Yoichi Watanabe

**Affiliations:** ^1^ Department of Diagnostic Radiology Hiroshima International University Higashi Hiroshima Hiroshima Japan; ^2^ Department of Radiation Oncology University of Minnesota Minneapolis MN USA

**Keywords:** cone‐beam CT, image quality, periodic QA, MTF, NPS

## Abstract

The image quality (IQ) of imaging systems must be sufficiently high for image‐guided radiation therapy (IGRT). Hence, users should implement a quality assurance program to maintain IQ. In our routine IQ tests of the kV cone‐beam CT system (Elekta XVI), image noise was quantified by noise standard deviation (NSD), which was the standard deviation of CT numbers measured in a small area in an image of an IQ test phantom (Catphan), and the high spatial resolution (HSR) was evaluated by the number of line‐pairs (LPN) visually recognizable in the image. We also measured the image uniformity, the low contrast resolution, and the distances of two points for geometrical accuracy. For this study, we did an additional evaluation of the XVI data for 12 monthly IQ tests by using noise power spectrum (NPS) for noise, modulation transfer function (MTF) for HSR, and CT number‐to‐density relationship. NPS was obtained by applying Fourier analysis in a small area on the uniformity test section of Catphan. The MTF analysis was performed by applying the Droege‐Morin (D‐M) method to the line‐pair bar regions in the phantom. The CT number‐to‐density relationship was obtained for insert materials in the low‐contrast test section of the phantom. All the quantities showed a noticeable change over the one‐year period. Especially the noise level improved significantly after a repair of the imager. NPS was more sensitive to the IQ change than NSD. MTF could provide more quantitative and objective evaluation of HSR. The CT number was very different from the expected CT number, but the CT number‐to‐density curves were constant within 5% except for two months. Since the D‐M method is easy to implement, we recommend using MTF instead of LPN even for routine QA. The IQ of the imaging systems was constantly changing; hence, IQ tests should be periodically performed. Additionally, we found the importance of IQ tests after every service work, including detector calibration as well as preventive maintenance.

PACS number(s): 87.56Da, 87.57.C‐, 87.57.N‐, 8757.Q‐

## I. INTRODUCTION

Cone‐beam computed tomography (CBCT) is routinely used for patient setup verification and position adjustment to deliver treatment with high precision for image‐guided radiation therapy (IGRT).^(1)^ The accuracy of this image‐based procedure heavily depends on the quality of the images obtained by the imaging tools. Hence, AAPM and other professional organizations strongly recommend periodic image quality (IQ) evaluation.^2^, ^3^ To meet the recommendation, physicists perform the IQ evaluation usually using a phantom specifically designed for such tests. We often evaluate IQ by measuring image noise, and low‐ and high‐contrast resolutions with simple methods, but there are more quantitative and accurate methods to evaluate the IQ of CBCT data.^4^, ^5^

We installed the first IGRT capable linear accelerator, Elekta Synergy (Elekta AB, Stockholm, Sweden), in 2007. The machine is equipped with an on‐board kV‐CBCT system, X‐ray volume imaging (XVI), and an MV electronic portal imaging system (EPID), iViewGT. To maintain the quality of the imaging tools, we established both a daily and monthly quality assurance (QA) program. For this study, we reanalyzed the XVI data for a one‐year period by using more quantitative physical quantities such as a noise power spectrum (NPS), a modulation transfer function (MTF), and CT number‐to‐density relationship. These quantitative IQ evaluation methods were compared with our standard IQ analysis methods. We expected that the more quantitative methods can minimize the observer dependence of the standard methods and maximize the information content of CBCT image data for better characterization of IQ. We found the usefulness of periodic IQ tests utilizing NPS and MTF. This study also showed the importance of IQ tests after any repair and maintenance service.

## II. MATERIALS AND METHODS

### A. CBCT quality assurance program

IQ evaluation methods were established based on published QA procedures recommended by national and international organizations.^2^, ^3^ Since the introduction of the Elekta Synergy in 2007, our IGRT‐related QA program has evolved and now it includes the coincidence test of the isocenter of the accelerator, kV‐CBCT, and EPID and IQ tests of 2D kV images. Current test items, as well as the tolerance levels, are summarized in Table 1.

**Figure 1:**
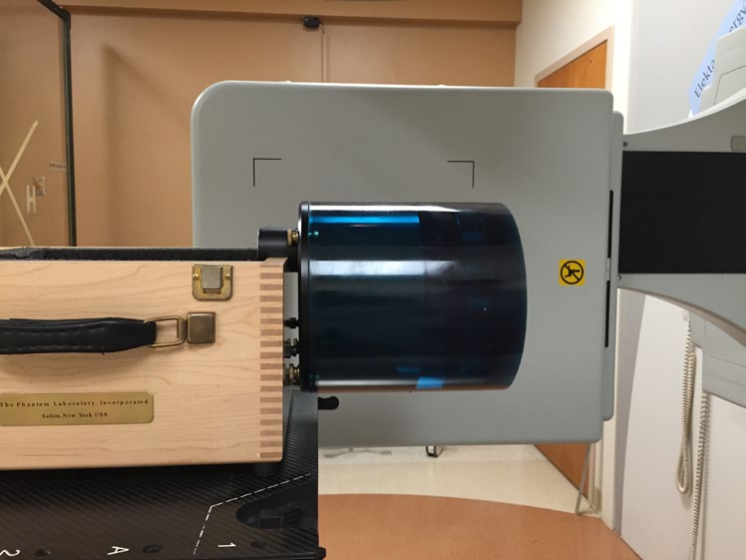
Photo of Catphan phantom for an image quality test.

We did monthly IQ tests of the XVI by scanning a Catphan503 phantom (The Phantom Laboratory, Greenwich, NY). Figure 1 shows the phantom mounted on the treatment couch for imaging. This phantom was made of three modules, CTP404, 486, and 528. Each module had a geometrical design and materials specific to the tests. CTP404 included eight cylindrical rods made of materials with different densities for measurement of CT number‐to‐density relationship. CTP486 was a water‐equivalent uniform phantom for testing the CT number uniformity and noise. CTP528 contained slits made of aluminum with different thickness and inter‐gaps for the high spatial resolution test. We scanned the phantom by using the most accurate, but the slowest imaging protocol available on the XVI system. This imaging protocol, Geometry calibration, used a full 360° rotation with the small field‐of‐view (FOV) setting and the S20 filter. The detailed imaging parameters are listed in Table 2. The imaging parameters used for clinical applications are different and those are specific to treatment sites. Table 2 shows also the imaging parameters for head‐and‐neck, pelvis, and thoracic areas. Therefore, it is noted that the IQ measured by our monthly QA was expected to be the highest achievable with the XVI system.

**Table 1:** Routine kV‐CBCT QA test items and tolerance used at our institution.

*Test Items*	*Daily*	*Monthly*	*Tolerance*
Safety: collision interlocks	X	X	Functional
Rotation center and machine isocenter coincidence	X	X	1.5 mm
CBCT image registration accuracy		X	1 mm
Couch movement accuracy after registration		X	1 mm
kV image and machine isocenter coincidence		X	1 mm
2D kV image quality: low contrast resolution		X	12 disks
2D kV image quality: high contrast resolution		X	10 line‐pair
CBCT image quality: uniformity and noise		X	2%/2%
CBCT image quality: low contrast resolution		X	2%
CBCT image quality: high spatial resolution		X	4 line‐pair^a^
CBCT image quality: geometrical accuracy		X	1.04 mm

a The baseline value at the time of installation was 7 line‐pair (lp)/cm, but later the tolerance level was set to a realistically achievable value of 4 lp/cm.

**Table 2:** Imaging parameters used for routine QA and clinical imaging on the XVI system on Elekta Synergy.

*Protocol*	*QA*	*Head&Neck*	*Chest*	*Pelvis*
Voltage (kVp)	120	100	120	120
Voxel size (mm)	0.5×0.5×2.5	1×1×2	1×1×3	1×1×3
Exposure (mAs)	1056	36.6	650	1056
Rotation angle	360	200	360	360
Filter types	S20, F0	S20, F0	M20, F0	M20, F0

Three‐dimensional volume image reconstruction was performed with the software on the machine. The voxel size of the raw data was $0.5\times 0.5\times 0.5\;{\text{mm}}^{3}$. To generate the data for the image analyses, we took an average of five slices. This resulted in $0.5\times 0.5\times 2.5\;{\text{mm}}^{3}$ as the voxel size of the reconstructed image. The reconstructed image data were then exported to image analysis software, ImageJ (National Institutes of Health, Bethesda, MD). Actual numerical calculations were performed by using the Microsoft Excel program (Microsoft Cooperation, Seattle, WA).

### B. Standard routine image quality tests

Our routine QA tests of the CBCT IQ included measurements of uniformity, noise, geometrical accuracy, low contrast resolution, and high spatial resolution. See Table 1 for the details.

The image uniformity and image noise were evaluated by measuring the CT number (CT#) in small areas on one of images inside CTP486. Four small ROIs (regions of interest) were created at the center of the circular phantom and the right, left, and anterior sides of the central ROI. The uniformity of the image was evaluated by calculating the uniformity index (UI) by taking the percentage difference between the maximum CT number among the average CT numbers measured in three peripheral areas $\left(\text{CT}{\#}_{\text{Max}}\right)$ and the average CT number of the central ROI $\left(\text{CT}{\#}_{\text{Center}}\right)$:^(6)^

(1) $$UI=100\frac{CT{\#}_{Max}-CT{\#}_{Center}}{CT{\#}_{Center}}$$

Noise standard deviation (NSD) was obtained by calculating the standard deviation of the CT numbers measured in the central ROI.

Low‐contrast resolution (LCR) was evaluated by measuring the average CT numbers of two insert materials, LDPE (Low Density Polyethylene) and Polystyrene (Poly), and calculating the low‐contrast visibility (LCV) given by the following vendor‐provided formula:^7^, ^8^

(2) $$LCV=\frac{2.75(CT\#{\left(Poly\right)}_{SD}+CT\#{\left(LPDE\right)}_{SD})}{CT\#{\left(Poly\right)}_{mean}-CT\#{\left(LPDE\right)}_{mean}}$$

High Spatial Resolution (HSR) was quantified by the number of line‐pairs (LPN) visually recognizable on a slice image inside CTP528.

### C. Quantitative image quality evaluation methods

#### C.1 Noise power spectrum (NPS)

**Figure 2:**
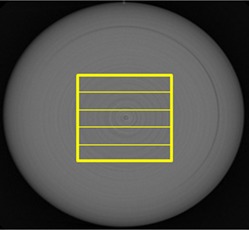
Slit ROIs used for NPS calculations.

We used the uniformity test section in the CTP486 module for this analysis. We created five rectangular ROIs (128 pixels × 15 pixels) shown in Fig. 2 and obtained the noise profile of each ROI.(910) Then, we calculated NPS_i_(f) of a frequency f for the i‐th ROI by using the formula:^4^, ^9^, ^11^

(3) $$NPS_{i}(f)=\frac{P\times P\times V}{N}\times {\left|F_{i}(f)\right|}^{2}$$

where *P* is the pixel size, *V* is the number of pixels along the horizontal direction in the ROI, *F_i_*(*f*) is the component for frequency *f* of the Fourier transform of the pixel data in the *i*‐th ROI, and *N* is the total number of pixels. Note that the background signal was approximated by using the fourth‐order polynomial to remove the nonuniformity of the image. Then, the space‐dependent CT numbers obtained from the equation were subtracted from the raw data before the Fourier analysis.

The final NPS was obtained by averaging the NPSs of five ROIs. To compare the NPS data with the standard noise parameter, NSD, we calculated an integrated NPS (iNPS) by integrating NPS over the entire spatial frequency range.

#### C.2 Modulation transfer function (MTF)

**Figure 3:**
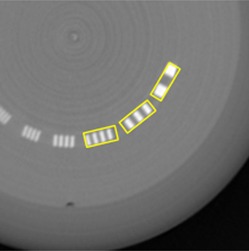
Rectangular ROI used for MTF calculations.

We used the high spatial resolution section in the CTP528 module. MTF was calculated by applying the Droege‐Morin (D‐M) method^(12)^ to the line‐pair bar regions in the module. The high resolution gauges in the phantom is made of 2 mm thick aluminum sheet with different width casted into epoxy. Those are arranged with specific gap width to create the line‐pair bars. The standard deviation (SD) of water, or epoxy for this phantom, ${\text{SD}}_{\text{water}}$, was obtained by measuring the CT numbers in the uniform region on the image; whereas the standard deviation of aluminum, ${\text{SD}}_{\text{Al}}$, was obtained by measuring the CT numbers inside the largest aluminum gauge on the image. To obtain the CT numbers and the SD value, ${\text{SD}}_{\text{line}-\text{pair}}$, we used a rectangular ROI on each line‐pair bar section as shown in Fig. 3. The D‐M formula of MTF was given by:

(4) $$MTF(f)=\frac{\pi \sqrt{2}}{4}\cdot \frac{M(f)}{M_{0}}$$

where

$$\begin{array}{l} M(f)=\sqrt{SD_{line\;pair}{\;}^{2}-SD^{2}}\\ SD^{2}=\frac{\left(SD_{Al}^{2}+SD_{water}^{2}\right)}{2}\\ M_{0}=\frac{\left|CT{\#}_{Al}-CT{\#}_{water}\right|}{2} \end{array}$$

Note that the spatial frequency f (cycles/mm) of a rectangular ROI is related to the number of line N by $f=\frac{N–1}{10}$

To compare HSR obtained by the number of visible line‐pairs, LPN, with the MTF, we introduced one spatial frequency, where the MTF value was 20% of the maximum value (or unity). This parameter was denoted by MTF_20%.

#### C.3 CT number versus density relation

**Figure 4:**
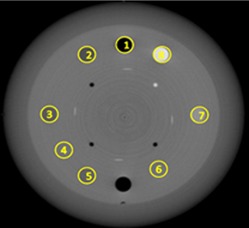
Eight ROIs used for CT number measurements.

We measured the CT numbers of eight inserts in the CTP404 module as shown in Fig. 4, where the numbers 1 to 8 indicate Air, PMP (polymethylpentene), LDPE (low‐density polyethylene), Epoxy, Polystyrene, Acrylic, Delrin, and Teflon, respectively.^(13)^ The material data are given in Table 3. The table also shows the CT numbers measured by using a 16‐slice Philips Brilliance Big Bore CT simulator (Philips Health Systems, Andover, MA) with its pelvis scanning protocol.

**Table 3:** Material data of the inserts in Catphan CTP404 module.^(13)^

*Insert No. in* Fig. 5	*Material*	*Effective Z*	*Specific Gravity*	*Relative Electron Density* ^a^	*CT Number* ^b^
1	Air	8.00	0.00	0.001	−830
2	PMP	5.44	0.83	0.853	−138
3	LDPE	5.44	0.92	0.945	−64
4	Epoxy	7.14	1.07	1.062	97
5	Polystyrene	5.70	1.03	0.998	−16
6	Acrylic	6.47	1.18	1.147	120
7	Delrin	6.95	1.42	1.363	308
8	Teflon	8.43	2.16	1.868	790

a Electron density relative to that of water.

b CT number obtained for a 16‐slice Philips Brilliance Big Bore CT simulator with the pelvis scanning protocol.

### D. Methods for evaluation of IQ parameters

The data collected for a 12‐month period were analyzed for the temporal changes of the IQ parameters. To investigate the usefulness of the IQ quantities of NPS and MTF, we plotted NSD vs. iNPS and LPN vs. MTF_20% as a function of time. The visual inspection of these graphs can provide the correlation between different IQ quantification methods. To do a more quantitative comparison, we applied statistical analysis methods to the same data set. Assuming a linear correlation between the noise parameters of NSD and iNSP, and the HSR parameters of LPN and MTF_20%, we computed the correlation coefficient, r, and the $R^{2}$ value of the linear regression. Note that a positive r‐value close to unity and a $R^{2}$ near unity imply a strong positive correlation between the IQ quantities obtained by different methods.

## III. RESULTS

### A. Image noise

**Figure 5:**
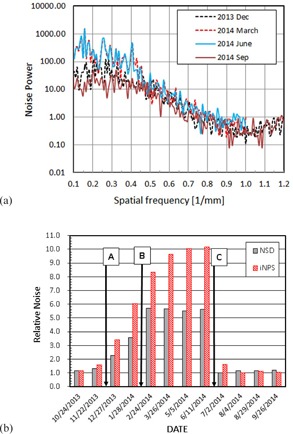
Image noise (a) NPS for 4 months, (b) monthly change of relative image noise. (A) Detector replacement on 12/21/13, (B) PM on 2/11/14, (C) Service on 6/12/14 and 6/17/14.

Figure 5 shows the monthly change of the image noise. In Fig. 5(a), the NPS functions are presented for four months, December 2013, March, June, and September 2014. The noise at low frequencies below 0.4 cycles/mm was larger by an order of magnitude for the months of March and June 2014 than those for December 2013 and September 2014, whereas all the curves showed a small difference in higher frequencies. Figure 5(b) shows NSD and iNPS for a one‐year period. Note that the noise quantities, NSD and iNPS, were normalized to the minimum value for each dataset. While there is a strong correlation between NSD and iNPS, $\left(r=0.9900\;\text{and}\;R^{2}=0.9803\right)$, iNPS showed clearly larger changes than NSD over this time period. It is noted that NSD was constant but iNPS kept increasing from February to June. The drop of the noise level after the detector calibration performed right before July 2nd QA (Table 4) is clearly observable by both methods.

**Table 4:** Summary of XVI service history.

*Date*	*Issue*	*Actions*
12/6/2013	Washed out image	Restarted the kV generator
12/16/2013	XVI CBCT was not working	Maybe SYNC cable problem
12/21/2013	XVI CBCT was not working	Replaced XVI detector
2/11/2014	No issue	Preventive maintenance (PM)
6/12/2014	Image artifact due to off position panel	□ Reestablished S‐M‐L panel position □ Calibrated panel movement □ Took Flex maps of S‐M‐L positions
6/17/2014	Image artifact	Performed XVI MLG (multilevel gain) calibration at 70 kV
9/23/2014	XVI panel beeps	Replaced XVI panel touch guard cable

### B. High spatial resolution (HSR)

**Figure 6:**
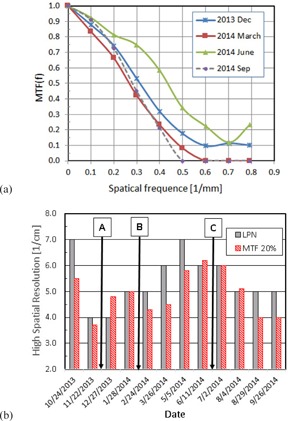
High spatial resolution (a) MTF for 4 months, (b) monthly change of high spatial resolution. (A) Detector replacement on 12/21/13, (B) PM on 2/11/14, (C) Service on 6/12/14 and 6/17/14.

Figure 6(a) shows the MTF curves obtained for months of December 2013, March, June, and September 2014. The data show that the magnitude of the MTF of March 2014, September 2014, December 2013, and June 2014 increased in this order in the entire frequency range. Figure 6(b) shows the monthly change of HSR using LPN and MTF_20% in the unit of cycles/cm. These two quantities were positively correlated $\left(r=0.9900\;\text{and}\;R^{2}=0.4654\right)$. Note that the LPN is an integer; whereas MTF_20% is a continuous variable. Hence, the latter enables us to detect smaller change of HSR than the former.

### C. Image uniformity and low contrast resolution

**Figure 7:**
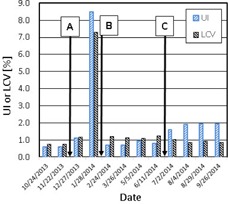
Image uniformity and low‐contrast resolution. (A) Detector replacement on 12/21/13, (B) PM on 2/11/14, (C) Service on 6/12/14 and 6/17/14.

Figure 7 shows the monthly variation of the image uniformity and the low‐contrast resolution as represented by UI and LCV, respectively. The tolerance values of the UI and LCV were 2%. The criteria were met (UI=0.60% to 1.95%, LCV=0.76% to 1.24%) except January 2014, which indicated very large UI and LCV of 8.5% and 7.3%. The low‐contrast resolution (LCR) (i.e., LCV) is an indicator of the signal‐to‐noise ratio (SNR); hence, a large LCV value is mainly due to a large noise. This can be easily understood from its definition given by Eq. (2). Figure 5(b) shows, in fact, that the noise in January 2014 was large, but not extreme in comparison to other months. Therefore, the very large LCV value of that month must be caused by other factors such as a change in the detector sensitivity.

### D. CT number‐to‐density relationship

**Figure 8:**
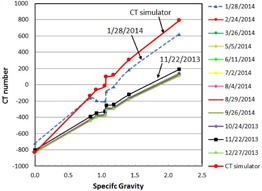
CT number‐to‐density relation.

Figure 8 shows the CT number‐to‐density relationship. The curves were constant within 5%, except two months, November 2013 and January 2014. The difference of November 2013 from the other months was about 10% and it was 20% or larger for January 2014. It is noted that the low‐contrast resolution (or LCV) measured on 1/28/2014 was very large (Fig. 7) and this corresponded to the abnormal CT numbers on that month. The CT number of XVI was about −220 for epoxy and it is very different from the common CT number. The difference can be clearly observed when the CT numbers of XVI and the Philips Brilliance CT are compared as shown in Table 3 and Fig. 8.

### E. XVI service and repair history

To understand the causes of the IQ changes, we examined the machine service record during the one‐year period for which the current QA data were collected. The documents were provided by service engineers after the work and those contained a brief description of the work performed for each service visit. Table 4 lists the service record. Major works which might have affected the IQ significantly included (A) replacement of the imager panel on December 21st, 2013, (B) preventive maintenance on February 11th, 2014, and (C) major detector calibration done on June 12th and 17th, 2014. The times of these services are indicated in Fig. 5(b), 6(b), and 7 to show the changes of the IQ parameters due to the work.

## IV. DISCUSSION

**Figure 9:**
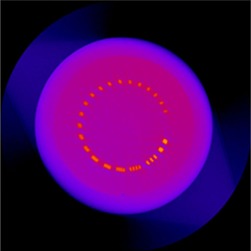
Image artifact observed in January 2014.

The IQ of the Elekta XVI system changed over a one‐year period. The changes were easily measurable by our IQ QA methods. The IQ improved after services by accelerator engineers. For example, the high noise level observed before July 2014 decreased considerably after the service for which the multilevel‐gain (MLG) calibration was done. This implied that the noise increases over a period of three to four months because the gain control of the XVI system deteriorates. The overall increase in the CT numbers observed in January 2014 was corrected by the preventive maintenance. In fact, the image of the phantom taken during our routine monthly QA on January 28th, 2014 showed noticeable artifacts, as shown in Fig. 9. It could be speculated that the artifacts were caused by a change in detector sensitivity, which resulted in poor detector calibration. In fact, the artifacts disappeared on the image taken at the next monthly QA tests, which were performed after the preventive maintenance on February 11. The same artifact was also a main reason for the very large image nonuniformity and LCV observed in January, as presented in Fig. 7. Our experience strongly suggests the value of routine maintenance along with close observation of the IQ through well‐designed QA procedures.

Both common IQ parameters such as NSD and LPN and the more quantitative parameters of iNSP and MTF_20% showed the variation of XVI IQ over a one‐year period. Hence, NSD and LPN can be used for routine CBCT IQ QA. NPS and MTF; in particular, the latter quantity can provide more objective and detailed information about the temporal variation of the characteristics of the imaging system. The calculation of NPS and MTF are rather straightforward with readily available software. There is an advantage of having NPS and MTF data over the data taken by the current standard techniques for routine monitoring of the IQ.

The frequency of IQ test of CBCT systems has not been established in the radiation oncology community. We observed that the IQ parameters usually made a gradual change with two months or longer time scale without service interventions. However, there was an exception in which the low‐contrast resolution suddenly worsened in a shorter time. Hence, we recommend monthly IQ QA. Additionally, it is worth emphasizing the need for IQ tests after every major service of the device.

The CT numbers of the Elekta XVI system are different from those expected for the standard CT system. One of the reasons is that the system is not calibrated to match the measured value with the standard CT numbers.^(14)^ The CT number‐to‐density relationship can be maintained within ±5% over a long period as long as the IQ is strictly monitored. Hence, the use of CT number for dose calculation for adaptive treatment may be possible with this system.

## V. CONCLUSIONS

All the IQ measures showed a noticeable change over a one‐year period. Especially, the noise level changed significantly after a repair of the imager. Furthermore, the NPS was more sensitive to the change of noise than NSD. MTF could provide more quantitative and observer‐independent evaluation of HSR.

We recommend using MTF instead of LPN even for routine periodic QA since the D‐M method is easy to implement and MTF is more objective. The periodic IQ test must be performed to catch a change of IQ, particularly after any modification of hardware and routine service.

## ACKNOWLEDGMENTS

Most of the work presented in this manuscript was performed while the first author was an exchange student visiting at University of Minnesota – Twin Cities. She was supported by the University of Minnesota‐Hiroshima International University Exchange Student Program. We would like to acknowledge Dr. Kozo Kumagai for his initiative in establishing this program and his continuing support. A part of this work was presented as an e‐poster (MO‐G‐CAMPUS‐J‐4) at the AAPM Annual Meeting in Anaheim, CA, on July 13th, 2015.

## COPYRIGHT

This work is licensed under a Creative Commons Attribution 4.0 International License.

## Supporting information

Supplementary Material
