# Supplementary figures and images for: One‐year analysis of Elekta CBCT image quality using NPS and MTF
Source: J Appl Clin Med Phys. 2016 May 8;17(3):211–22. doi: 10.1120/jacmp.v17i3.6047 (PMC5690923; doi:10.1120/jacmp.v17i3.6047)

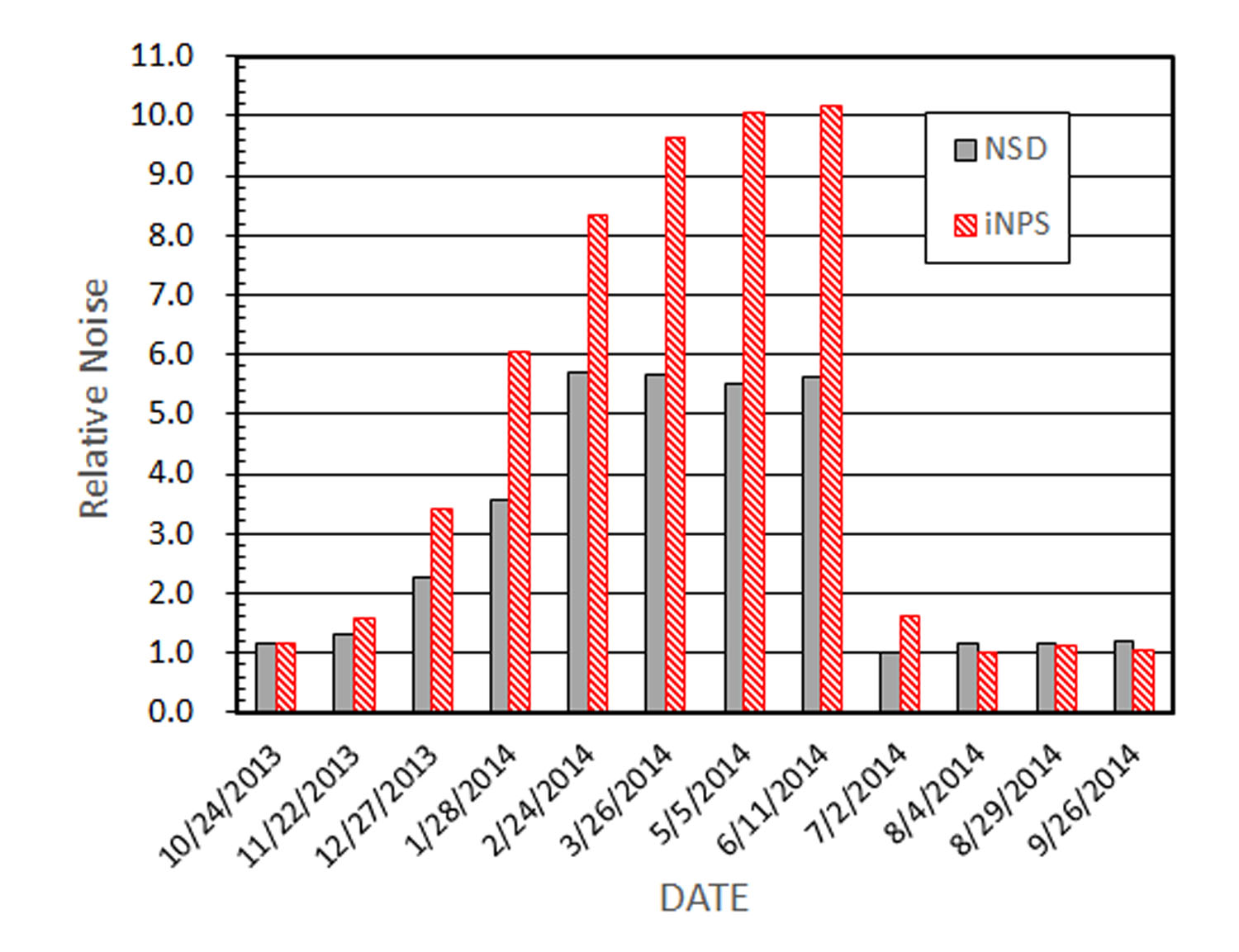

Supplement: Supplementary file 1 — Supplementary Material [file ACM2-17-211-s001.jpg]
